# Physiotherapy Approach to a Bilateral Pontine Infarct With Acute Ischemic Stroke: A Case Report

**DOI:** 10.7759/cureus.55046

**Published:** 2024-02-27

**Authors:** Shraddha S Kochar, Snehal Samal

**Affiliations:** 1 Neuro Physiotherapy, Ravi Nair Physiotherapy College, Datta Meghe Institute of Higher Education and Research, Wardha, IND

**Keywords:** rehabilitation, acute ischemic stroke, pontine infarct, case report, physiotherapy

## Abstract

Strokes are prevalent, although only some impact the pons, a brainstem region that performs essential functions. Because pontine infarcts are uncommon, there isn’t sufficient research to back up efficient therapies that give patients functional advantages. This case report presents a 75-year-old male who was brought to the casualty with a history of falls and developed weakness on the left side of the body, dysarthria, and dysphagia, for which he was referred to the neurophysiotherapy department after performing the necessary investigations. A neurological examination was carried out, and a physiotherapy protocol was made. Outcome measures were taken before and after physiotherapy rehabilitation, including the Glasgow Coma Scale (GCS), Berg Balance Scale, and Functional Independence Measure (FIM). The physiotherapy treatment showed a considerable improvement in the patient’s overall health.

## Introduction

One of the most severe diseases endangering people’s neurological health is stroke, which is the third most prevalent cause of death after cancer and cardiovascular disease because of its high recurrence rate, morbidity, and mortality [1]. The pons, which is the most significant structure of the brainstem and is situated proximal to the medulla oblongata and distal to the midbrain, is the exact location of any acute or chronic interruption of blood supply that results in pontine infarction, a kind of ischemic stroke [2]. Approximately 15% of acute vertebrobasilar ischemic strokes and 7% of all ischemic infarctions are caused by pontine infarction, which is more common than any other isolated brainstem infarct [3]. Acute ischemic stroke (AIS) is a condition characterized by an abrupt interruption of blood supply to a specific region of the brain, which results in an impairment in neurological abilities [4]. Additionally, women who are considered at a higher risk during the perimenopausal period and into later age groups have a higher incidence of AIS [5]. Because the risk of an ischemic stroke rises with age, an elderly individual with a history of chronic diseases, such as diabetes, hypertension, dyslipidemia, or cardiovascular disease, is more inclined to be diagnosed with a pontine infarction [6]. Diagnosing pontine infarctions can be difficult and can lead to a wide range of symptoms. These can include reduced consciousness, respiratory and cardiac malfunction, the uncommon locked-in syndrome, the classical crossing syndrome, and the less common pure motor hemiparesis/hemiplegia or pure sensory stroke [7].

The clinical presentation of a pontine infarct depends on the anatomical/arterial territories involved. Bilateral pontine infarcts cause blockage in the larger basilar artery’s blood flow [6]. Physiotherapy plays a very vital role in managing patients with pontine infarct having AIS. The primary objective of physical therapy is to restore as many of the functions that the patient has lost as possible and to facilitate the restoration of functions that have been severely and permanently destroyed [8].

## Case presentation

Patient information

A 75-year-old male farmer by occupation was brought to the casualty with a history of giddiness, due to which he experienced a fall from a height of approximately 7 feet at his home in the morning five days back. Since the fall, he started complaining of weakness in his left upper and lower extremities, slurring of speech, and difficulty in swallowing. However, as the symptoms aggravated, the patient came to the casualty, where the history was taken. The patient was advised to undergo interventions like a complete blood count, liver function test, kidney function test, and magnetic resonance imaging (MRI) of the brain. The patient has a known case of hypertension and has been on antihypertensive drugs for eight years. The patient was prescribed medicine and directed to neurophysiotherapy for additional management concerning all of the previously mentioned complaints, which the patient’s relative reported.

Clinical findings

After obtaining consent from the patient, a neurological examination was carried out. The patient was conscious and cooperative, with the Glasgow Coma Scale (GCS) score being E4V1M4, which showed moderate brain injury, with the patient not responding to verbal commands. Upon observation, the patient had a mesomorphic body type. He was lying supine, and on measuring vitals, his blood pressure was 145/90 mmHg, and his heart rate, respiratory rate, and oxygen saturation were normal. Upon examination, all the cranial nerves were intact. Upon sensory assessment, all the senses were present. Tables 1, 2 show muscle tone (tone grading system) findings of the patient’s left upper and lower extremities and reflexes. The muscle tone of the right side of the body was normal, which was +2 (normal tone). He needed moderate assistance for sitting, but for standing and walking, he was entirely dependent and needed maximum assistance. The Berg Balance Score was 10, which is a high risk of falls.

**Table 1:** Muscle tone of left upper and lower extremities 1+: decreased tone; 2+: normal tone; 3+: increased tone

Muscles	Left upper and lower extremities
Shoulder	
Flexors	1+
Extensors	1+
Abductors	1+
Adductors	1+
Elbow	
Flexors	1+
Extensors	1+
Hip	
Flexors	1+
Extensors	1+
Abductors	1+
Adductors	1+
Knee	
Flexors	1+
Extensors	3+
Ankle	
Plantarflexors	1+
Dorsiflexors	1+

**Table 2:** Motor examination (reflexes) +: diminished reflex; ++: normal reflex; +++: exaggerated reflex

Reflexes	Right	Left
Biceps	++	+
Triceps	++	+
Knee	++	+++
Ankle	++	+
Plantar response	Flexor	Mute

Investigations

**Figure 1:**
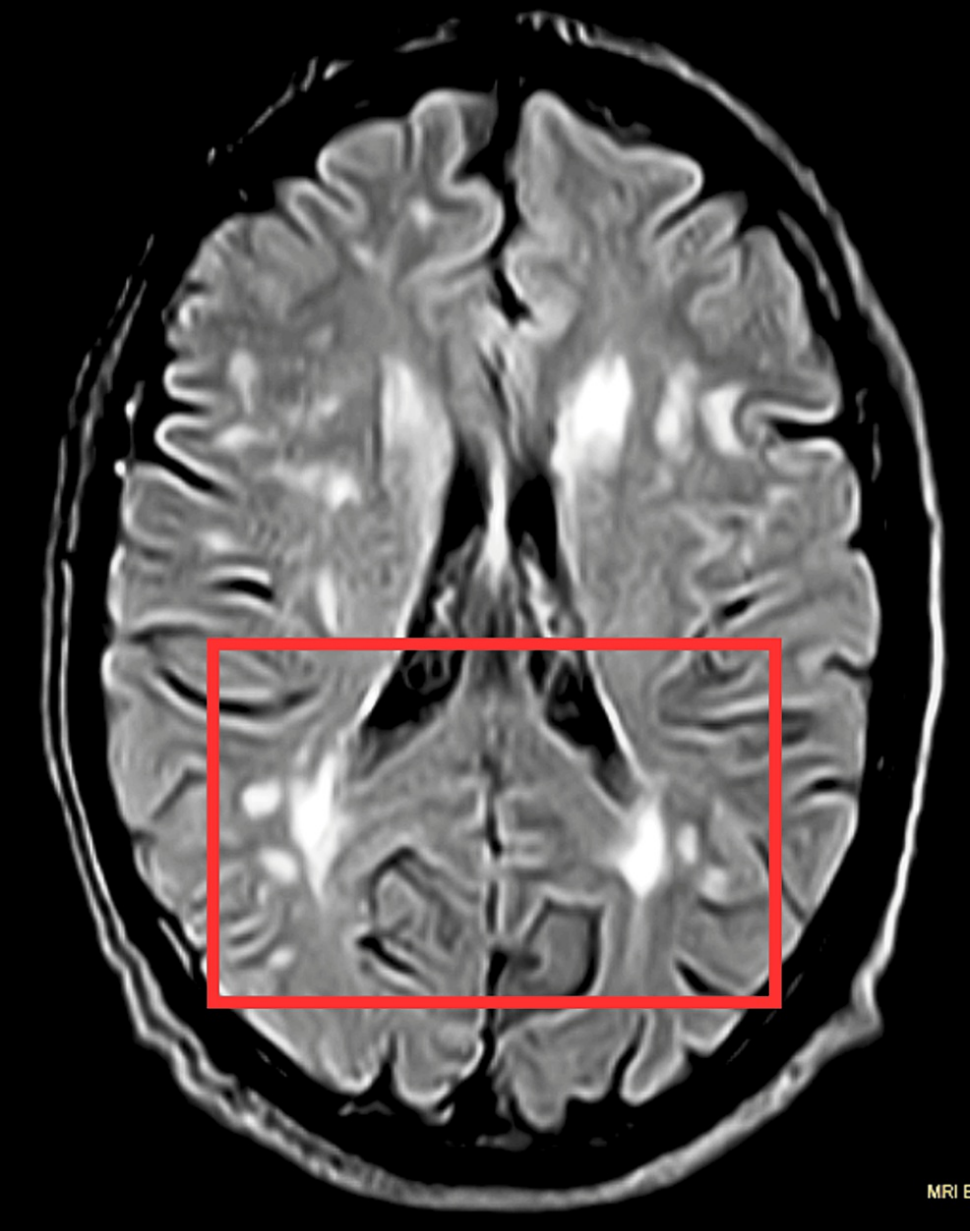
MRI brain The red rectangle represents bilateral pontine infarct. MRI, magnetic resonance imaging

An MRI of the brain revealed an altered signal intensity area in the left gangliocapsular region. The prominence of sulcogyral spaces, cerebellar folia, and ventricular system suggested age-related changes. The impression of the MRI brain also revealed a bilateral pontine infarct with acute infarct in the left gangliocapsular region and chronic lacunar infarct in the right external capsule. Figure 1 shows MRI-brain findings.

Physiotherapy management

Table 3 shows the physiotherapy intervention received by the patient over a span of two weeks to improve his health condition.

**Table 3:** Physiotherapy intervention ROM, range of motion; PNF, proprioceptive neuromuscular facilitation; D, diagonal; reps, repetitions; BOS, base of support; CTAR, chin tuck against resistance

Goals	Intervention	Dosage
Patient and relative education	The patient's condition and the advantages and importance of physiotherapy were explained. In addition, the treatment's potential to prevent problems and enhance his ability to walk, climb stairs, and perform other daily tasks was described to him and his relatives.	N/A
To improve the flexibility of muscles and ROM	PNF rhythmic initiation for the left side upper and lower limb (D1 and D2 patterns) was given to the patient in supine position along with joint approximation. The intervention then progressed to PNF, a combination of isotonic and slow reversals.	10 reps x 1 set
To enhance the strength of muscle	Strengthening exercises were given to the patient for all the four limbs. One liter bottle was used to strengthen the shoulder, elbow, and wrist muscles of the right side, and a half-liter bottle for the left side, which was performed by the patient with mild assistance. Dynamic quads for the right leg and static quads for the left leg were given for quadriceps strengthening.	10 reps x 1 set; this was progressed to 10 seconds of hold.
To improve bed mobility	The transition from supine to side lying to sitting was given to the patient. This also reduced the chances of developing bed sores (positioning given).	Every 2 hours
To enhance balance	Progression from standing to standing on wide BOS to narrow BOS, and as the patient developed some amount of balance, the training progressed to tandem standing to tandem walking.	The exercises progressed as the patient gained balance, and each position was held for 30 seconds initially, and then the time was increased to 1 minute.
To improve gait	Spot marching was given to the patient, which then progressed to hallway walking and walking within the parallel bars by placing the mirror in front of the patient to get the biofeedback, thereby improving the gait of the patient.	Thrice a day
To improve speech	The patient was asked to say the vowels out loudly while sitting in front of the mirror so that the patient could get the feedback of himself. This was progressed to melodic intonation therapy, which involved humming, sighing, or singing out in an intoned phrase(s) the requests, feelings, or contributions in communication, which was followed by verbal exchange therapy in which the patient’s close ones were asked to talk with the patient so that the patient initiates the talk.	Twice a day
To improve ability to swallow	Orofacial physiotherapy was given, which consisted of facial muscle strengthening exercises and tongue exercises. Exercises that work directly on hyoid musculature were also given, which included Shaker maneuver and CTAR.	10 reps x 1 set, twice a day

Follow-up and outcome measures

The patient visited the neuro rehabilitation unit three weeks after discharge for a rehabilitation follow-up. At the end of his treatment, the assessment was done again. The patient reported improvement in the strength of the left side of the body with improved standing balance and walking and decreased fall risk, and his ability to swallow was improving with improvement in speech. The tone of the left side of the body showed improvement, with reflexes being normal. GCS showed mild brain injury post-treatment, the Berg Balance Scale showed that the patient had a medium risk of fall, and Functional Independence Measure (FIM) showed he needed only minimal assistance in performing the activities. The pre- and post-treatment outcomes are given in Table 4.

**Table 4:** Outcome measures GCS, Glasgow Coma Scale; FIM, Functional Independence Measure; E, eye response; V, verbal response; M, motor response

Outcome measures	Pre-treatment	Post-treatment
Sitting	Moderate support needed	Sits independently
Standing	Full assistance needed	Stands with minimal assistance
Walking	Full assistance needed	Minimal assistance needed
GCS	E4V1M4	E4V4M5
Berg Balance Scale	10/56	40/56
FIM	52	84

## Discussion

In the above case, we saw the physiotherapy rehabilitation of a patient who was having bilateral pontine infarct with AIS. Our patient-tailored physiotherapy protocol showed significant progress after five weeks of treatment. The literature on physiotherapy intervention in bilateral pontine infarct with AIS is minimal. A study conducted by Flores demonstrated that a 75-year-old female suffering from a left-sided pontine infarct was managed with physiotherapy. The protocol consisted of gait training, transfer training, lower extremity strengthening, and balance training, which benefited this patient in regaining functional movement [9]. In the study conducted by Chaturvedi et al., proprioceptive neuromuscular facilitation patterns were given to the patient to improve the muscle’s flexibility and increase the joint’s range of motion. This treatment showed an improvement and reduced the weakness in the body [10].

A study was done by Konecny et al., in which they used orofacial physiotherapy for dysphagia after stroke. The treatment was given for eight weeks and showed a significant improvement in the swallowing and food intake process of the patients with difficulty swallowing [11]. The research was published that consisted of various speech therapeutic protocols that could help the patient improve their speech. The protocol included coughing therapy, verbal expressive training, picture exchange therapy, vocal play exercises, melodic intonation therapy, verbal exchange therapy, and music therapy [12]. As suggested by Smithard, exercises that worked directly on the strengthening of hyoid musculature were given, like Shaker maneuver, chin tuck against resistance (CTAR), VitalStim, and Ampcare, which showed a great effect in enhancing the ability of the individual to swallow [13]. Shaker maneuver and CTAR were given to the patient mentioned in this report and showed a significant improvement in the patient’s swallowing ability.

## Conclusions

For patients suffering from bilateral pontine infarction with AIS, physiotherapy plays a very essential role. Pontine infarcts are not recognized at the early stage, so it becomes important for the patients to recognize the symptoms as early as possible. This case report describes an old male patient with a bilateral pontine infarct with AIS who was referred to neurophysiotherapy for the management of the complaints. The patient reported an increase in ROM, enhanced muscle strength, reduced difficulty in swallowing, ability to speak in a less slurred voice, and increased overall balance, which resulted in proper gait. As there is very little research on the physiotherapy treatment of this condition, this case report contributes vital information to already available information. 
